# The application of L‐glutaminase for the synthesis of the immunomodulatory *γ*‐D‐glutamyl‐L‐tryptophan and the kokumi‐imparting *γ*‐D‐glutamyl peptides

**DOI:** 10.1002/fsn3.1845

**Published:** 2020-09-23

**Authors:** Juan Yang, Yuran Huang, Hao Dong, Guiying Huang, Limei Yu, Weidong Bai, Xiaofang Zeng

**Affiliations:** ^1^ College of Food Science and Technology Zhongkai University of Agriculture and Engineering Guangzhou China; ^2^ Academy of Contemporary Agricultural Engineering Innovations Zhongkai University of Agriculture and Engineering Guangzhou China

**Keywords:** enzymatic synthesis, glutaminase, *γ*‐D‐glutamyl dipeptides, *γ*‐D‐glutamyl‐L‐tryptophan (SCV‐07)

## Abstract

Glutaminase of *Bacillus amyloliquefaciens* has been used to synthesize the immunomodulatory *γ*‐D‐glutamyl‐L‐tryptophan (*γ*‐D‐Glu‐L‐Trp) and the kokumi‐active *γ*‐D‐glutamyl peptides. The optimum yield of *γ*‐D‐Glu‐L‐Trp was 55.76 mM in corresponding to a minimum yield of by‐product (*γ*‐D‐Glu‐*γ*‐D‐Glu‐L‐Trp) in the presence of 75 mM D‐Gln and 100 mM L‐Trp. The glutaminase has a low K_m_ values for the donors (D‐Gln and L‐Gln:5.53 and 0.98 mM), but high ones for the acceptors (L‐Trp, L‐Phe, L‐Met, L‐Val and *γ*‐[D‐Glu]_(_ *_n_*_=1,2,3)_‐L‐Val/L‐Phe/L‐Met, ranging from 32.51 to 193.05 mM). The highest K_m_ value appearing when *n* = 2 (*γ*‐[D‐Glu]_(_ *_n_*_=0,1,2)_‐L‐Val/L‐Phe/L‐Met) suggested the rising difficulty for synthesis when the number of donor increases in the reaction mixtures. The *γ*‐[D‐Glu]_(_ *_n_*_=1,2,3)_‐L‐Val/L‐Phe/L‐Met at 5 mM can impart the blank chicken broth an enhancing monthfulness, thickness, and umaminess taste.

## Graphical abstract

Glutaminase of Bacillus amyloliquefaciens has been used to synthesize the immunomodulatory γ‐D‐glutamyl‐L‐tryptophan (γ‐D‐Glu‐L‐Trp) and the kokumi‐active γ‐D‐glutamyl peptides. The optimum yield of γ‐D‐Glu‐L‐Trp was 55.76 mM in corresponding to a minimum yield of byproduct (γ‐D‐Glu‐γ‐D‐Glu‐L‐Trp) in the presence of 75 mM D‐Gln and 100 mM L‐Trp. The glutaminase has a low Km values for the donors (D‐Gln and L‐Gln:5.53 and 0.98 mM), but high ones for the acceptors (L‐Trp, L‐Phe, L‐Met, L‐Val and γ‐[D‐Glu](n = 1,2,3)‐L‐Val/Phe/Met, ranging from 32.51 to 193.05 mM). The highest Km value appearing when n=2 (γ‐[D‐Glu](n = 0,1,2)‐L‐Val/Phe/Met) suggested the rising difficulty for synthesis when the number of donor increases in the reaction mixtures. The γ‐[D‐Glu](n = 1,2,3)‐L‐Val/Phe/Met at 5 mM can impart the blank chicken broth an enhancing monthfulness, thickness, and umaminess taste.

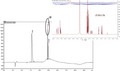

## 1 INTRODUCTION

*γ*‐Glutamyl transpeptidation reaction catalyzed by *γ*‐glutamyltranspeptidase (GGT, EC 2.3.2.2) or certain L‐glutaminases (L‐Gln amidohydrolase EC 3.5.1.2) mainly involves a *γ*‐glutamyl donor (L‐Gln or D‐Gln) providing the *γ*‐glutamyl moiety and a *γ*‐glutamyl acceptor (other amino acids and peptides) linking with the acquired *γ*‐glutamyl group, thus, *γ*‐D‐ or *γ*‐L‐glutamyl peptides are synthesized selectively (Nandakumar, Yoshimune, Wakayama, & Moriguchi, 2003; Suzuki et al., 2003; Suzuki, Yamada, & Kato, 2007). Although both L‐Gln and D‐Gln can be used as the *γ*‐glutamyl donor for providing the *γ*‐glutamyl group, the difference is D‐Gln can not be as an acceptor to form the by‐products like *γ*‐D‐Glu‐D‐Gln and other poly‐D‐glutamyl peptides so that the yield of *γ*‐D‐glutamyl dipeptide is significantly higher than that of *γ*‐L‐glutamyl peptide when GGT was as the catalysis in the transpeptidation reaction (Morelli, Calvio, Biagiotti, & Speranze, 2014; Nandakumar et al., 2003). For example, the yield of *γ*‐glutamyl‐taurine dramatically increased from 25% (*γ*‐L‐Glu‐Tau) to 71% (*γ*‐D‐Glu‐Tau), since *γ*‐D‐Glu‐D‐Gln and *γ*‐D‐Glu‐*γ*‐D‐Glu‐Tau were not synthesized (Suzuki, Miyakama, & Kumagai, 2002; Suzuki et al., 2007).

Although the production of by‐products would be reduced with the D‐Gln as *γ*‐glutamyl donor, the practical application of D‐Gln for the synthesis of *γ*‐D‐glutamyl peptides is rare except for *γ*‐D‐Glu‐L‐Trp (SCV‐07) which was mainly synthesized using GGT as the catalyst in the presence of D‐Gln and L‐Trp (Saini, Bindal, & Gupta, 2017; Suzuki, Kato, & Kumagai, 2004). SCV‐07, as an immunomodulator peptide, is often used as a therapeutic to attenuate the severity and duration of both acute and fractionated radiation‐induced oral mucositis in a clinical trial of head and neck cancer patients (Adkins et al., 2010; Alterovitz, Tuthill, Rios, Modelska, & Sonis, 2011; Watkins, Pouliot, Fey, Tuthill, & Sonis, 2010); inhibit growth of a variety of tumor cell line types, including in leukemia, lymphoma, herpes simplex virus type 2, head and neck cancer xenograft models (Papkoff et al., 2011; Tuthill, Paploff, Watkins, & Sonis, 2011, Ii et al. 2008), as well as stimulate the differentiation of T‐lymphocyte and the expression of Thy1‐antigen on bone marrow cells, and enhance the production of interleukin 2 and interferon in mice (Simavirtsev et al., 2003).

Recently, the glutaminase from *Bacillus amyloliquefaciens* has been used as a catalysis to synthesize a series of kokumi‐imparting *γ*‐[L‐Glu]_n_‐Phe/Val/Met/Tyr short chains peptides with the synthetic *γ*‐[L‐Glu]_(n‐1)_‐AA as the acceptors (Yang, Bai, Zeng, & Cui, 2019; Yang, Sun‐Waterhous, Cui, Zhao, & Dong, 2018; Yang, Sun‐Waterhous, Xie, et al., 2018; Yang, Sun‐WaterhousE, Cui, Dong, & Wer, 2017). The biggest characteristic of the transpeptidation by this glutaminase‐catalysis is the production of multiple by‐products like *γ*‐[L‐Glu]2‐Val/Phe/Met/Tyr, *γ*‐[L‐Glu]3‐Val/Phe/Met/Tyr and *γ*‐[L‐Glu]4‐Val/Phe/Met/Tyr. Although these by‐products were coined as kokumi‐imparting peptides, the yield of the “so called” product, *γ*‐glutamyl dipeptides, was seriously restricted. Whether D‐Gln can be as a *γ*‐glutamyl donor to reduce the production of by‐products, as well as for the synthesis of *γ*‐D‐Glu‐L‐Trp is worth trying. Therefore, the glutaminase form *Bacillus amyloliquefaciens* was used as a catalysis to synthesize *γ*‐D‐Glu‐L‐Trp and various *γ*‐D‐glutamyl peptides.

## 2 MATERIAL AND METHODS

### 2.1 Reagent and enzyme

Commercial synthetic *γ*‐D‐Glu‐L‐Trp, *γ*‐D‐Glu‐*γ*‐D‐Glu‐L‐Trp, *γ*‐L‐Glu‐L‐Trp, *γ*‐L‐Glu‐*γ*‐L‐Glu‐L‐Trp, *γ*‐D‐Glu‐L‐Phe, *γ*‐D‐Glu‐L‐Met and *γ*‐D‐Glu‐L‐Val were obtained from Apeptides (Shanghai, China). D‐Gln, L‐Gln, L‐Val, L‐Trp, L‐Met, L‐Phe, Gly‐Gly, and *γ*‐glutamyl‐p‐nitroanilide (*γ*‐GpNA) were purchased from Sigma‐Aldrich (Shanghai, China). L‐Glutaminase of *Bacillus amyloliquefaciens* was acquired from Amano Enzyme China Ltd. (Shanghai, China). The HPLC grade acetonitrile and formic acid were purchased from CapitalBio Corporation (shanghai, China).

### 2.2 Enzymatic synthesis of *γ*‐D‐glutamyl peptides

: Synthesis of *γ*‐D‐Glu‐L‐Trp: a reaction mixture that contained D‐Gln (100 mM, pH 10.0), L‐Trp (100 mM, pH 10.0), and glutaminase (0.05 U/ml) was placed in a water bath shaking table for incubating for 8 hr at 37 ºC, and then the mixture was inactivate enzyme in a boiling water bath at 90 ºC for 15 min. The optimum ratio of substrate was also analyzed including the various D‐Gln (75, 100, 150 mM) and a fixed L‐Trp (100 mM). The isolation of *γ*‐D‐Glu‐Dipeptides was carried in accordance with the method of Suzuki with some modification (Dong, Li et al., 2020; Dong, Xian, et al., 2020; Suzuki et al., 2004). A 50 ml reaction mixture was applied to a column (2.3 × 7.2 cm) of Dowex 1 × 8 that was prepared as the HCOO‐ form. The column was washed with 0.5% formic acid aqueous solution for eluting the *γ*‐D‐Glu‐L‐Trp. The fractions only containing *γ*‐D‐Glu‐L‐Trp were collected and lyophilized using a freeze dryer (Scientz‐N, Ningbo, China), and then purified *γ*‐D‐Glu‐L‐Trp was dissolved in D2O and then analyzed with a Bruker 600 MHz Superconducting Fourier Transform NMR Spectrometry (AVANCE III HD 600, Switzerland). HPLC (Model L‐7100; Hitachi, Tokyo, Japan) equipped with XSelect HSS T3 column (5 μm 4.6 × 250 mm; Waters, USA) was used to determine the content of peptides and the L‐Trp. Acetonitrile and water with 0.1% formic acid were as the mobile phase. The other parameter contained: 1 ml/min flow rate, 220 nm detection wavelength (λ), 40°C column temperature, and 20 μL injection volume. The objective products in the HPLC chromatogram were preliminarily identified on the basis of the retention time of external standard compounds.
: Synthesis of several kinds of *γ*‐D‐Glutamyl peptides: The reaction mixture contained one of the substrate mixtures of D‐Gln:L‐Met = 75:100, D‐Gln:L‐Val = 75:100 or D‐Gln:L‐Phe = 75:100, 0.05 U/ml enzyme, incubated in a water bath shaking table at 37°C for 12 hr, then the mixtures were heated to the point of inactivating enzyme in a boiling water bath at 90 ºC for 15 min. These *γ*‐D‐Glu‐peptides in the mixtures were analyzed by UPLC‐Q‐TOF MS/MS system (Agilent 1,290) with an Agilent ZORBAX RRHD SB‐C18 (2.1 × 50 mm, 1.8 μm) 110 column, referring to Yang's methods (Yang, Sun‐Waterhous, Cui, et al., 2018; Yang, Sun‐Waterhous, Xie, et al., 2018; Yang et al., 2017).

Enzymatic kinetic parameters (K_m_) of glutaminase for the synthesis of *γ*‐D‐glutamyl peptides.

The apparent K_m_ of the acceptors for the synthesis of *γ*‐D‐glutamyl peptides were determined through analyzing the concentration of the products via UPLC‐MS/MS. The concentration of substrates was range 1 ~ 25 mM, the K_m_ and V_max_ values were calculated by the Michaelis–Menten equation. Moreover, the K_m_ of D‐Gln and L‐Gln were calculated through measuring the content of *γ*‐D/L‐Glu‐L‐Trp at the various D/L‐Gln concentration (1 ~ 25 mM) and a fixed L‐Trp (25 mM).

The substrate specificity of the enzyme with different acceptors were measured as described previously with some modification (Shuai, Zhang, Mu, & Jiang, 2011). Briefly, an enzymatic reaction condition was as follows: all the *γ*‐GpNA, *γ*‐glutamyl acceptors and enzyme in the borate‐NaOH (50 mM, pH 10.0) buffer solution, then adding 0.1 M HCl to terminate the reaction after incubation at 37 ºC for 30 min. The content of *p*‐nitroaniline was determined by a UV spectrophotometer (UV765, Shanghai, China) at 410 nm. The release of 1 μmol of p‐nitroaniline per min from *γ*‐GpNA was defined as the amount of one unit (U) of enzyme through the transpeptidation reaction.

Preliminary sensory evaluation of *γ*‐D‐glutamyl dipeptides.

5 mM of these synthetic *γ*‐D‐glutamyl peptides (*γ*‐[L‐Glu]n‐L‐Phe/L‐Met/L‐Val) were added into a blank chicken broth to evaluate the flavor characteristic. Taste profiles mainly included mouthfulness, thickness and umaminess. Thereinto, mouthfulness describes an overall perception associated with food texture, structure and morphological complexity, and thickness which was measured as the increased taste intensity evaluated 10 s after food tasting describes rich complexity. The panels were consisted of 10 males and 7 females (age 30–40) from a professional company. 10 ml of solutions was given to these panelist individually and then put into the mouth for 15 s before swallowing, finally recorded the taste experienced in 25 s. Panelists were asked to rate the intensity of a given taste on a scale from 0 (not detectable) to 5 (strongly detectable).

### 2.3 Statistical analysis

Statistical analysis was performed in triplicate and analyzed by Microsoft Excel 2000. Duncan test was used to determine significant differences at *p* < .05 between samples using SPSS 16.0 statistical software.

## 3 RESULTS AND DISCUSSION

### 3.1 The enzymatic synthesis of *γ*‐D‐glutamyl peptides

*γ*‐D‐Glu‐L‐Trp.

**FIGURE 1:**
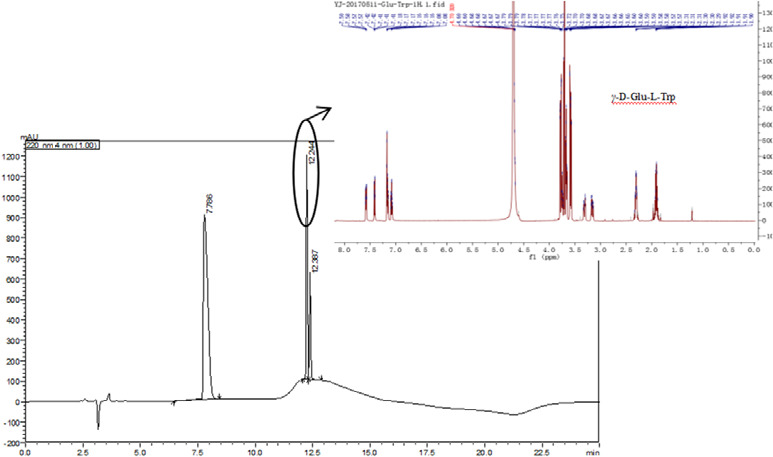
A HPLC chromatogram (λ = 220 nm) containing L‐Trp (7.786 min), *γ*‐D‐Glu‐L‐Trp (12.244 min) and *γ*‐D‐Glu‐*γ*‐D‐Glu‐L‐Trp (12.387 min), inserted NMR spectra of the *γ*‐D‐Glu‐L‐Trp

Three main intense chromatographic peaks were observed, the most intense peak corresponded to *γ*‐D‐Glu‐L‐Trp (12.245 min) indicating that *γ*‐D‐Glu‐L‐Trp could be enzymatically synthesized in the action of a commercial glutaminase of *Bacillus amyloliquefaciens* (Figure 1). The product is collected from the fraction and the NMR spectra of the *γ*‐D‐Glu‐L‐Trp were identified by NMR. *γ*‐D‐Glu‐L‐Trp (1H NMR, 600 MHz, D_2_O): δ 7.61−7.55 (m, 1H), 7.41 (dq, J = 8.2, 1.2 Hz, 1H), 7.19−7.13 (m, 2H), 7.08 (ddq, J = 8.1, 7.0, 1.2 Hz, 1H), 4.71−4.64 (m, 4H), 3.81−3.72 (m, 4H), 3.74−3.69 (m, 3H), 3.67 (ddd, J = 8.8, 6.1, 2.8 Hz, 3H), 3.59 (ddd, J = 11.8, 6.2, 0.8 Hz, 3H), 3.31 (dt, J = 14.8, 4.4 Hz, 1H), 3.16 (ddd, J = 14.7, 8.3, 2.8 Hz, 1H), 2.35−2.27 (m, 2H), 1.95−1.87 (m, 2H).

**FIGURE 2:**
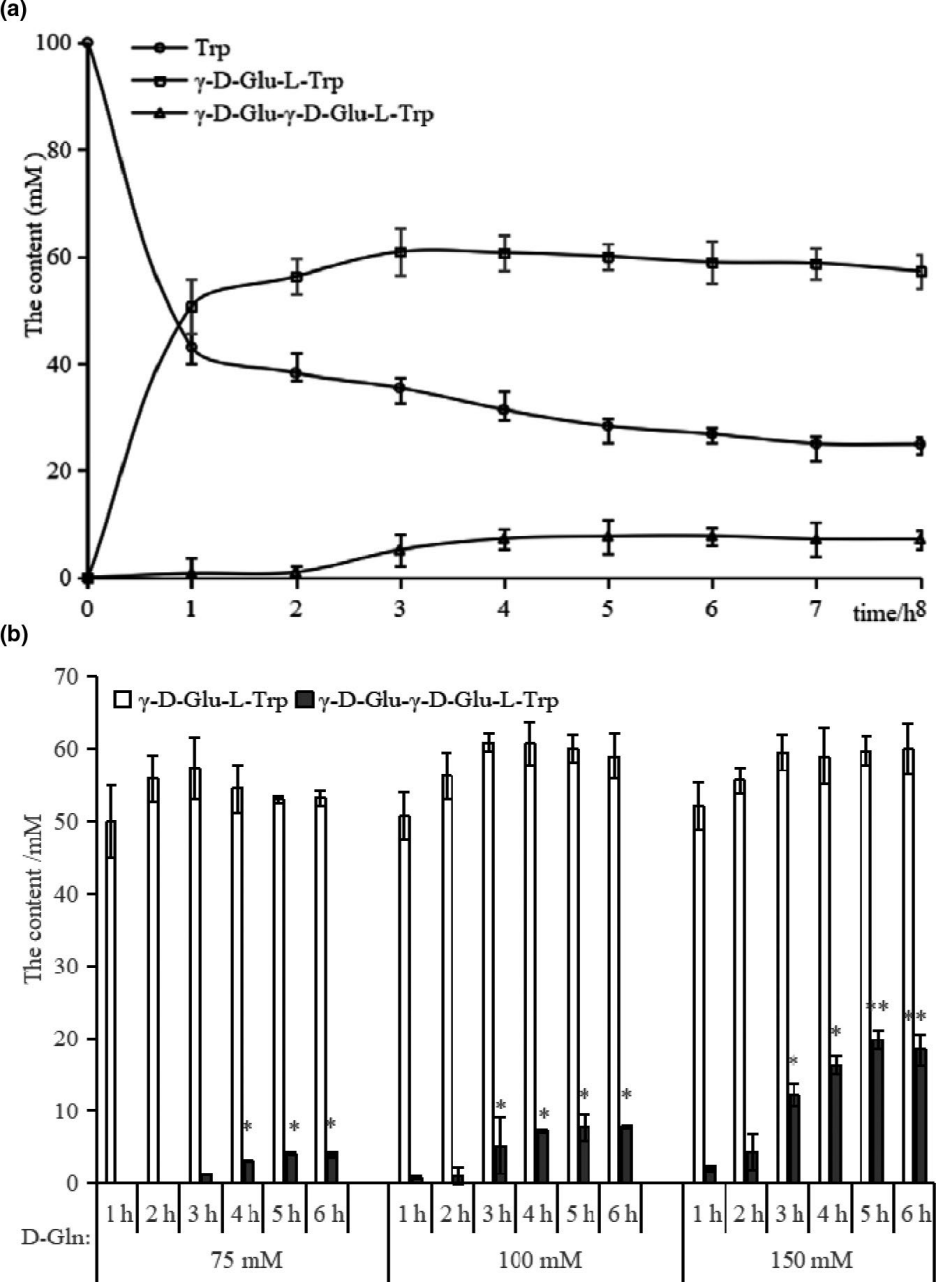
(a) Glutaminase‐catalyzed synthesis of *γ*‐D‐Glu‐L‐Trp, corresponding consumption of Trp and the by‐product (*γ*‐D‐Glu‐*γ*‐D‐Glu‐L‐Trp) as a function of reaction time. The reaction mixture for synthesis (pH 10.0) contain equimolar amounts (100 mM) of D‐Gln and L‐Trp, L‐glutaminase (0.05 U/ml) and incubated at 37°C for 8 hr, determined by HPLC. (b) The effect of D‐glutamine on the *γ*‐D‐Glu‐L‐Trp synthesis. The mixture for synthesis (pH 10.0): a fixed L‐Trp (100 mM), various D‐Gln (75, 100, 150 mM), L‐glutaminase (0.05 U/mL) and incubated at 37°C for 6 hr

The other two intense peaks were the L‐Trp (7.762 min) and *γ*‐D‐Glu‐*γ*‐D‐Glu‐L‐Trp (12.389 min), suggesting that there was a significant coexisting product (*γ*‐D‐Glu‐*γ*‐D‐Glu‐L‐Trp) in the postenzymatic reaction mixture. The production of *γ‐*D‐Glu‐L‐Trp and *γ*‐D‐Glu‐*γ*‐D‐Glu‐L‐Trp, as well as the consumption of L‐Trp with time‐course are showed in Figure 2a. The objective *γ‐*D‐Glu‐L‐Trp would reach a maximum yield of 60.90 mM after 3 hr incubation (its yield increasing during the first 3 hours but without significant differences) and then leveled off, the result was closed to that of GGT‐catalyzing (Suzuki et al., 2004). Meanwhile, the by‐product, *γ*‐D‐Glu‐*γ*‐D‐Glu‐L‐Trp, was about 1 mM yield during the first 2 hours of incubation, and then dramatically increased to 7.72 mM during the next 3 hr (from the 2nd to 5th h), finally maintained stability (*p* > .05). The observation illustrates there was a time difference between obtaining the maximum of *γ‐*D‐Glu‐L‐Trp (3 hr of incubation) and *γ*‐D‐Glu‐*γ*‐D‐Glu‐L‐Trp (5 hr), so the concentration of by‐product can be minimized through controlling the incubation time. Correspondingly, the consumption of L‐Trp can be summarized in roughly three stages during the synthesis of *γ*‐D‐Glu‐L‐Trp: firstly, L‐Trp sharply dropped to 43.12 mM after 1 hr reaction, then dropped at a lower rate from the 1st to 5th h, and finally reached a plateau (L‐Trp at about 28.36 mM) after the 5th h (i.e., differences were statistically insignificant).

When comparing the sequence characteristic between *γ*‐D‐Glu‐L‐Trp and *γ*‐D‐Glu‐*γ*‐D‐Glu‐L‐Trp (a 2‐fold difference of glutamic acid residue), it can be speculated the concentration of D‐Gln would influence the production of by‐product more than that of objective product. Therefore, the effect of D‐Gln (75, 100, 150 mM) with a fixed L‐Trp (100 mM) was analyzed for reducing the yield of *γ*‐D‐Glu‐*γ*‐D‐Glu‐L‐Trp (Figure 2b). With a fixed incubation time, it can be observed the yield of *γ*‐D‐Glu‐*γ*‐D‐Glu‐L‐Trp was influenced positively by the concentration of D‐Gln, while its effect on the *γ*‐D‐Glu‐L‐Trp yield seems to be negligible. For which the lower concentration of D‐Gln should be selected in the *γ*‐D‐Glu‐L‐Trp synthesis. Generally, the reaction condition can be set to 75 mM D‐Gln and 100 mM L‐Trp, incubating for 2 hr at 37°C (pH 10.0). The yield of *γ*‐D‐Glu‐L‐Trp was 55.85 mM, with a below detectable limit of *γ*‐D‐Glu‐*γ*‐D‐Glu‐L‐Trp.

γ‐D‐Glutamyl Phenylalanine, Methionine, and Valine.

The objective *γ*‐D‐Glu‐L‐Phe, *γ*‐D‐Glu‐L‐Val, and *γ*‐D‐Glu‐L‐Met were with the significant signals at *m/z* 295.1295, 247.1299, and 279.1016, respectively in positive ESI mode (Table 1). Their fragments mainly contained [M‐AA(Acceptor)‐COOH]^+^ (*m/z* = 84, b‐CO_2_ ion), [AA(Acceptor)‐COOH]^+^ (y‐CO_2_ type ion), [AA(Acceptor)‐NH_3_]^+^ (y‐CO_2_ type ion), [AA(Acceptor)+H]^+^ (y type ion) in each of the mass spectrum. Their yield was 30.23 ± 4.18, 23.12 ± 3.97, 17.19 ± 2.04 mM, respectively (Table 2), accounting for 117.90%, 115.43%, and 120.63% of their L‐configurations in the same reaction condition (L‐configurations’ synthesis have been reported by Yang). The result indicated the yield of *γ*‐glutamyl dipeptides slightly increased with D‐Gln as a *γ*‐glutamyl donor instead of L‐Gln in *γ*‐glutamyl peptides synthesis, which was in line with the reports (Suzuki et al., 2002, 2003).

**TABLE 1:** Ions of parent and fragment, proposed γ‐D‐glutamyl peptides sequences in the post‐enzymatic mixtures

Substrates/Acceptor	Parent ion(*m/z*)	Product ions by ESI MS/MS(*m/z*)	Proposed *γ*‐D‐glutamyl peptides
L‐Trp	334.1460	188.0712 [Trp‐NH_3_ + H]^+^, 84.0447 [M‐Trp‐COOH]^+^	*γ*‐D‐Glu‐L‐Trp
	463.1823	334.1395, 188.0705, 84.044	*γ*‐[D‐Glu]_2_‐L‐Trp
L‐Phe	295.1295	166.0860 [Phe + H]^+^, 120.0810 [Phe‐COOH]^+^, 84.0446 [M‐Phe‐COOH]^+^	*γ*‐D‐Glu‐L‐Phe
	424.1721	295.1288, 166.0861, 120.0807, 84.0444	*γ*‐[D‐Glu]_2_‐Phe
	553.2141	424.1692, 295.1277, 166.0859, 84.0443	*γ*‐[D‐Glu]_3_‐Phe
L‐Val	247.1299	84.0452 [M‐Val‐COOH]^+^, 72.0817 [Val‐COOH]^+^	*γ*‐D‐Glu‐L‐Val
	376.1735	230.1029, 184.0977, 118.0868, 72.0817	*γ*‐[D‐Glu]_2_‐L‐Val
	505.2164	376.1719, 247.1297, 184.0972, 118.0867, 72.0816	*γ*‐[D‐Glu]_3_‐L‐Val
L‐Met	279.1016	150.0577 [Met + H]^+^, 133.0317, [Met‐NH_3_ + H]^+^, 84.0448[M‐Met‐COOH]^+^	*γ*‐D‐Glu‐L‐Met
	408.1446	279.1013, 150.0583, 133.0319, 104.0531 [Met‐COOH]^+^, 84.0447	*γ*‐[D‐Glu]_2_‐L‐Met
	537.1877	408.1434, 279.1010, 150.0584, 133.0500, 104.0532, 84.0446	*γ*‐[D‐Glu]_3_‐L‐Met

**TABLE 2:** The yield difference between *γ*‐D‐glutamyl peptides and *γ*‐L‐glutamyl peptides

*γ*‐D‐glutamyl peptides	Content (mM)	*γ*‐L‐glutamyl peptides	Content (mM)
*γ*‐D‐Glu‐L‐Phe	30.23 ± 4.18	*γ‐*L‐Glu‐L‐Phe	25.64 ± 1.11
*γ*‐[D‐Glu]_2_‐Phe	5.89 ± 0.56	*γ*‐[L‐Glu]_2_‐L‐Phe	9.66 ± 0.85
*γ*‐[D‐Glu]_3_‐Phe	2.12 ± 0.78	*γ*‐[L‐Glu]_3_‐L‐Phe	5.54 ± 0.54
*‐*	‐	*γ*‐[L‐Glu]_4_‐L‐Phe	1.75 ± 0.42
*‐*	‐	*γ*‐[L‐Glu]_5_‐L‐Phe	0.13 ± 0.052
*γ*‐D‐Glu‐L‐Val	23.12 ± 3.97	*γ*‐L‐Glu‐L‐Val	20.03 ± 2.48
*γ*‐[D‐Glu]_2_‐L‐Val	6.23 ± 1.03	*γ*‐[L‐Glu]_2_‐L‐Val	10.36 ± 1.07
*γ*‐[D‐Glu]_3_‐L‐Val	1.35 ± 0.97	*γ*‐[L‐Glu]_3_‐L‐Val	6.07 ± 0.92
*‐*	‐	*γ*‐[L‐Glu]_4_‐L‐Val	3.33 ± 0.57
*γ*‐D‐Glu‐L‐Met	17.19 ± 2.04	*γ*‐L‐Glu‐L‐Met	14.25 ± 1.42
*γ*‐[D‐Glu]_2_‐L‐Met	9.79 ± 1.22	*γ*‐[L‐Glu]_2_‐L‐Met	13.06 ± 1.84
*γ*‐[D‐Glu]_3_‐L‐Met	3.94 ± 0.89	*γ*‐[L‐Glu]_3_‐L‐Met	7.11 ± 0.87
*‐*	‐	*γ*‐[L‐Glu]_4_‐L‐Met	2.75 ± 0.52

Note

The reaction mixture for *γ*‐D‐/L‐Glutamyl peptides synthesis (pH 10.0) set as the optimal concentration of references, and the transpeptidation reaction was carried out at pH 10.0 and 37 ºC, 0.05 U/ml glutaminase, the concentration of *γ*‐D‐/L‐Glutamyl peptides were measured as materials and methods.

The by‐products including *γ*‐[D‐Glu]_2_‐Phe and *γ*‐[D‐Glu]_3_‐Phe, *γ*‐[D‐Glu]_2_‐Val and *γ*‐[D‐Glu]_3_‐Val, *γ*‐[D‐Glu]_2_‐Met and *γ*‐[D‐Glu]_3_‐Met were detected in each reaction mixtures, and there content were 5.89 ± 0.56 and 2.12 ± 0.78, 6.23 ± 1.03 and 1.35 ± 0.97, 9.79 ± 1.22 and 3.94 ± 0.89 mM, respectively (Table 2)). When L‐Gln as a *γ*‐glutamyl donor instead of D‐Gln, the number and content of by‐products were increased. Namely, the *γ*‐[L‐Glu]_4_‐Phe and *γ*‐[L‐Glu]_5_‐Phe, *γ*‐[L‐Glu]_4_‐Val and *γ*‐[L‐Glu]_4_‐Met were detected only when L‐Gln was as the *γ*‐glutamyl donor. The content of *γ*‐D‐glutamyl oligopeptides was 22.24%~74.96% of that of *γ*‐L‐glutamyl oligopeptides. These results indicated the by‐products would be still synthesized with a decreasing number of by‐products and the production of by‐products. In other words, unlike GGT, glutaminase can catalyze *γ*‐D‐glutamyl di‐/tri‐peptide as the acceptor to synthesize the short‐chain *γ*‐D‐glutamyl oligopeptides (Suzuki et al., 2002, 2003). In addition, the *γ*‐D‐Glu‐D‐Gln and other poly‐D‐glutamylated species were not detected, indicating D‐Gln can not be an acceptor to participate in a *γ*‐glutamyl transfer reaction by glutaminase‐catalyzing.

Comparison of the affinities of glutaminase for *γ*‐D‐glutamyl peptides.

According to the multiple products and the catalytic properties of glutaminase, the synthetic route of the products is the synthetic *γ*‐D‐[Glu]_(n‐1)_‐L‐AA as an acceptor to participate in the *γ*‐glutamyl transfer reaction to form the by‐products (*γ*‐D‐[Glu]_n_‐L‐AA) (Yang, Sun‐Waterhous, Cui, et al., 2018; Yang, Sun‐Waterhous, Xie, et al., 2018; Yang et al., 2017). However, the number of the products and their content with the D‐Gln as the acceptor was less than that of L‐Gln in the same reaction condition. It seems to indicate that *γ*‐D‐[Glu]_n_‐L‐AA has a low affinity for this enzyme. Therefore, the affinity of these *γ*‐D‐[Glu]_n_‐L‐AA to this enzyme and the differences of the affinity of the L‐ and D‐ configurations to this enzyme has been determined.

The results of the Km values of glutaminase show high Km values for acceptors, but low ones for *γ*‐glutamyl donors, (Table 3). The Km values for D‐Gln and L‐Gln (the donors) were 5.53 and 0.98 mM, indicating glutaminase has the highest affinity for L‐Gln. These values are higher than that of GGT from *Bacillus Atrophaeusdonors*, *Bacillus licheniformis*, *Bacillus Subtilis*, and *Bacillus pumilus* (Saini et al., 2017), but be in close proximity to that of GGT of *Bacillus subtilis SK11.004* (Shuai et al., 2011). The K_m_ values for the acceptors were range from 32.51 to 193.05, whose magnitude is in accordance with that of GGT of *Bacillus subtilis* SK11.004 (Shuai et al., 2011). The K_m_ values for the acceptors (*γ‐*[D‐Glu]_(_ *_n_*_=0,1,2)_‐L‐Trp/Phe/Met/Val) during *γ*‐glutamyl transpeptidation by glutaminase‐catalysis were getting bigger with the increased “n,” that is, the lowest K_m_ with L‐Trp/L‐Phe/L‐Met/L‐Val (*n* = 0, 32.51, 46.30, 88.81 and 76.63 mM), while the highest (67.49, 135.23, 165.36 and 193.05 mM) with *γ‐*[D‐Glu]_(_ *_n_*_=1)_‐L‐Trp, *γ‐*[D‐Glu]_(_ *_n_*_=2)_‐L‐Phe, *γ‐*[D‐Glu]_(_ *_n_*_=2)_‐L‐Met and *γ‐*[D‐Glu]_(_ *_n_*_=2)_‐L‐Val. Thus, it was more and more difficult for the synthesis of *γ‐*[D‐Glu]n‐L‐Trp/L‐Phe/L‐Met/L‐Val became more difficult with the increase of the number of *γ*‐D‐glutamyl residues in the acceptor, which were consistent with *γ‐*[L‐Glu]n‐L‐Phe/L‐Met/L‐Val synthesis. The K_m_ values of *γ‐*[D‐Glu]_(_ *_n_*_=1,2,3)_‐L‐Trp/L‐Phe/L‐Met/L‐Val during the hydrolysis reaction by glutaminase‐catalysis were in the range of 36.73 ~ 122.73 mM, which were lower than those for the synthesis of *γ‐*[D‐Glu]_n_‐L‐Trp/L‐Phe/L‐Met/L‐Val. Moreover, the affinities of glutaminase for the same acceptors with the L‐Gln as the donor were obviously lower than that of D‐Gln. The affinities of glutaminase for the acceptors illustrate L‐Trp is the most natural acceptor, indicating the glutaminase is very suitable for the synthesis of *γ‐*D‐Glu‐L‐Trp.

**TABLE 3:** The affinities of glutaminase (K_m_, mM) for the synthesis and hydrolysis of different L‐amino acid and *γ*‐D‐glutamyl peptides with a fixed D‐Gln

	Synthesis	Hydrolysis		Synthesis	Hydrolysis
D‐Gln*	5.53 ± 0.98	‐	L‐Gln*	0.98 ± 0.08	‐
L‐Trp	32.51 ± 3.86	‐	L‐Trp	20.54 ± 1.01	‐
*γ*‐D‐Glu‐L‐Trp	67.49 ± 4.43	36.73 ± 2.86	*γ*‐L‐Glu‐L‐Trp	27.44 ± 1.43	15.71 ± 1.82
*γ*‐[D‐Glu]_2_‐Trp	‐	96.44 ± 3.25	*γ*‐[L‐Glu]_2_‐L‐Trp	182.93 ± 7.00	26.41 ± 1.05
			*γ*‐[L‐Glu]_3_‐L‐Trp	232.71 ± 10.26	42.71 ± 3.06
			*γ*‐[L‐Glu]_4_‐L‐Trp	‐	75.13 ± 4.67
L‐Phe	46.30 ± 2.32	‐	L‐Phe^a^	47.88 ± 0.47	‐
*γ*‐D‐Glu‐L‐Phe	89.44 ± 6.31	52.81 ± 3.02	*γ‐*L‐Glu‐L‐Phe^a^	84.89 ± 1.02	24.81 ± 1.02
*γ*‐[D‐Glu]_2_‐Phe	135.23 ± 7.81	71.33 ± 2.05	*γ*‐[L‐Glu]_2_‐L‐Phe^a^	95.23 ± 7.05	30.73 ± 2.05
*γ*‐[D‐Glu]_3_‐Phe	‐	106.42 ± 2.05	*γ*‐[L‐Glu]_3_‐L‐Phe^a^	126.47 ± 10.05	56.42 ± 2.05
			*γ*‐[L‐Glu]_4_‐L‐Phe^a^	206.47 ± 13.05	70.79 ± 3.86
			*γ*‐[L‐Glu]_5_‐L‐Phe^a^	‐	80.75 ± 3.11
L‐Met	88.81 ± 6.12	‐	L‐Met^b^	42.92 ± 3.48	‐
*γ*‐D‐Glu‐L‐Met	87.76 ± 7.97	62.19 ± 4.61	*γ*‐L‐Glu‐L‐Met^b^	37.76 ± 8.27	30.16 ± 2.05
*γ*‐[D‐Glu]_2_‐L‐Met	165.36 ± 4.28	82.48 ± 6.75	*γ*‐[L‐Glu]_2_‐L‐Met^b^	53.17 ± 5.24	42.48 ± 2.75
*γ*‐[D‐Glu]_3_‐L‐Met	‐	102.73 ± 5.26	*γ*‐[L‐Glu]_3_‐L‐Met^b^	122.23 ± 6.19	72.73 ± 5.96
			*γ*‐[L‐Glu]_4_‐L‐Met^b^	‐	105.13 ± 9.01
L‐Val	76.63 ± 3.25	‐	L‐Val^b^	53.49 ± 1.84	‐
*γ*‐D‐Glu‐L‐Val	127.45 ± 6.76	68.78 ± 5.25	*γ*‐L‐Glu‐L‐Val^b^	97.44 ± 5.02	29.76 ± 2.85
*γ*‐[D‐Glu]_2_‐L‐Val	193.05 ± 8.24	90.41 ± 6.25	*γ*‐[L‐Glu]_2_‐L‐Val^b^	113.07 ± 8.04	66.41 ± 2.75
*γ*‐[D‐Glu]_3_‐L‐Val	‐	122.73 ± 8.72	*γ*‐[L‐Glu]_3_‐L‐Val^b^	142.44 ± 10.23	82.71 ± 3.56
			*γ*‐[L‐Glu]_4_‐L‐Val^b^	‐	95.15 ± 5.11

Note

The kinetic parameters for the transpeptidation substrates L‐amino acids (1 ~ 25 mM) with a fixed D‐Gln (25 mM) were calculated by Michaelis–Menten equation through measuring the content of *γ*‐D‐glutamyl compounds via HPLC. D‐Gln and L‐Gln were determined via the synthesis of *γ*‐D‐Glu‐L‐Trp.

* Donors; a: the reference (Yang et al., 2017), b: the reference (Yang, Sun‐Waterhous, Xie, et al., 2018).

In addition, the substrate specificity of the glutaminase for the transpeptidase activity was also determined and the result revealed that various amino acids could serve as *γ*‐glutamyl acceptors with the D‐Gln as the *γ*‐glutamyl donor (Table 4). Besides Gly‐Gly control, aromatic amino acids (L‐Try, L‐Phe, L‐Tyr, and L‐Met), L‐His, L‐Val, and L‐Asn were good acceptors; L‐Ala, L‐Gly, L‐Glu, L‐Gln, L‐Pro, and L‐Ser were poor acceptors. The enzyme exhibited similar specificity with GGT from *Bacillus subtilis* SK11.004 in catalyzing L‐amino acids as *γ*‐glutamyl acceptors (Shuai et al., 2011).

**TABLE 4:** Substrate specificity of glutaminase for *γ*‐Glutamyl acceptors with the *γ*‐D‐GpNA

Substrate	Reactive act. (%)	Substrate	Reactive act. (%)
L‐Ser	‐	L‐Phe	70.00 ± 3.98
L‐Gly	13.41 ± 1.14	L‐Gln	2.93 ± 0.08
L‐Pro	5.61 ± 0.18	L‐Cys	44.39 ± 1.02
L‐Ala	8.54 ± 0.98	L‐Asn	80.49 ± 4.20
L‐Thr	43.90 ± 4.39	L‐Leu	50.24 ± 2.22
L‐Ile	34.39 ± 2.35	L‐Met	105.61 ± 6.30
L‐Val	80.00 ± 3.24	L‐Trp	98.42 ± 5.39
L‐His	68.05 ± 5.01	L‐Glu	‐
L‐Lys	41.46 ± 0.52	L‐Asp	31.71 ± 3.22
L‐Arg	35.12 ± 1.23	L‐Tyr	42.93 ± 4.18

Note

Transferase activity was measured by a spectrophotometric method as described in Materials and Methods. Activity is expressed relative to that found with 20 mM Gly‐Gly (100%).

Sensory characteristics of individual *γ*‐[D‐Glu]n‐L‐Phe/Met/Val.

**FIGURE 3:**
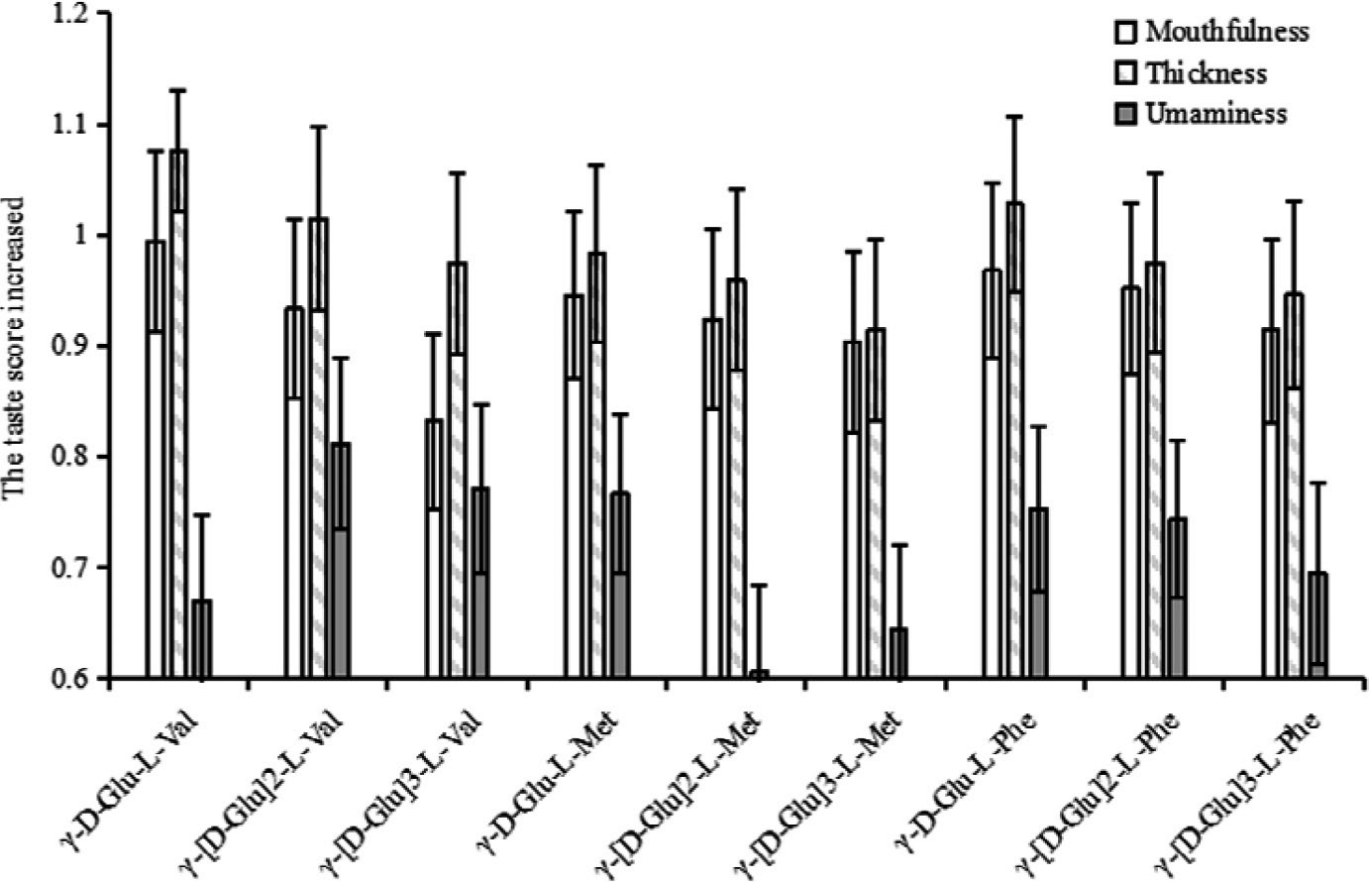
Profiles of enhanced sensory attributes by *γ‐*[L‐Glu]n‐L‐Phe/L‐Met/L‐Val. All the *γ‐*[L‐Glu]n‐L‐Phe/L‐Met/L‐Val were dissolved in model chicken broth (MCB), the pH is adjusted to 6.5

It is known that the series short‐chain *γ‐*[L‐Glu]n‐L‐Phe/L‐Met/L‐Val exhibited kokumi properties and can enhance the thickness, mouthfulness, and umaminess of model chicken broth (Yang et al., 2017, 2019; Yang, Sun‐Waterhous, Xie, et al., 2018). Therefore, the kokumi characteristics of these *γ*‐D‐Glutamyl peptides were analyzed, and the results showed the addition of *γ‐*[D‐Glu]n‐L‐Phe/L‐Met/L‐Val at 5 mM to a chicken broth caused significant (*p* < .05) an enhancing mouthfulness, thickness and umami taste with the taste‐enhancing score of 0.60 ~ 1.07 (Figure 3). The enhancing taste intensities by these *γ*‐D‐Glutamyl peptides was lower than that of *γ*‐L‐Glutamyl peptides, for instance, the taste‐enhancing score of the umaminess, thickness, and mouthfulness intensity by *γ‐*[L‐Glu]n‐L‐Phe at 5 mM were ranging from 0.95 ~ 1.41(Yang et al., 2017). Actually, it has been reported that D‐configuration and L‐configuration have the same flavor characteristics like the umami‐active D‐Theanine and L‐Theanine (Suzuki et al., 2003).

## 4 CONCLUSIONS

*γ*‐D‐glutamyl peptides including the immunomodulatory peptide (*γ*‐D‐Glu‐L‐Trp) and *γ*‐[D‐Glu]n‐L‐Phe/L‐Met/L‐Val have been synthesized in the presence of D‐Gln catalyzed by the glutaminase of *B. Amyloliquefaciens*. Differ from the GGT enzyme, this glutaminase can catalyze the binding of D‐Gln to the synthetic *γ*‐D‐glutamyl di‐/tri‐peptides. When comparing the D‐Gln and L‐Gln as the donors, the number and content of the by‐products with the D‐Gln as the donor were significantly less than that of L‐Gln in the *γ*‐glutamyle peptides synthesis. The K_m_ values of glutaminase of *B. Amyloliquefaciens* for D‐Gln and L‐Gln (the donors) were 5.53 and 0.98 mM, and for the acceptors were range from 32.51 (L‐Trp) to 193.05 mM (*γ*‐[D‐Glu]_2_‐L‐Val). The optimal yield of *γ*‐D‐Glu‐L‐Trp was 55.85 mM with a below detectable limit of *γ*‐D‐Glu‐*γ*‐D‐Glu‐L‐Trp under the reaction condition: 75 mM D‐Gln and 100 mM L‐Trp, incubating for 2 hr at 37°C (pH 10.0). The addition of the *γ*‐[D‐Glu]n‐L‐Phe/Met/Val can impart the blank chicken broth an enhancing kokumi taste.

## CONFLICT OF INTEREST

The authors declare that they do not have any conflict of interest.

## ETHICAL APPROVAL

This study does not involve any human or animal testing.
